# Ugonin P mitigates osteolytic bone metastasis by suppressing MDK via upregulating miR-223-3p expression

**DOI:** 10.7150/ijbs.111356

**Published:** 2025-05-31

**Authors:** Yat-Yin Law, Haritha Rengamanar, Chih-Ying Wu, Chih-Chuang Liaw, Shubham Suresh Ghule, Yu-Ying Wu, Kuan-Ying Lai, Le Huynh Hoai Thuong, Trung-Loc Ho, Athena Yanjen Lin, Yi-Chin Fong, Chun-Hao Tsai, Chih-Hsin Tang

**Affiliations:** 1 School of Medicine, Chung Shan Medical University, Taichung, Taiwan; 2 Department of Orthopedics, Chung Shan Medical University Hospital, Taichung, Taiwan; 3 Graduate Institute of Biological Science and Technology, China Medical University, Taichung, Taiwan; 4 Department of Neurosurgery, China Medical University Hospital, Taichung, Taiwan; 5 Department of Neurosurgery, China Medical University Hsinchu Hospital, Hsinchu, Taiwan; 6 Department of Marine Biotechnology and Resources, National Sun Yat-sen University, Kaohsiung, Taiwan; 7 Graduate Institute of Natural Products, Kaohsiung Medical University, Kaohsiung, Taiwan; 8 Graduate Institute of Biomedical Sciences, China Medical University, Taichung, Taiwan; 9 Institute of Medicine, Chung Shan Medical University, Taichung, Taiwan; 10 Taipei American School, Taipei, Taiwan; 11 Department of Sports Medicine, College of Health Care, China Medical University, Taichung, Taiwan; 12 Department of Orthopedic Surgery, China Medical University Hospital, Taichung, Taiwan; 13 Department of Orthopedic Surgery, China Medical University Beigang Hospital, Yunlin, Taiwan; 14 Department of Pharmacology, School of Medicine, China Medical University, Taichung, Taiwan; 15 Chinese Medicine Research Center, China Medical University, Taichung, Taiwan; 16 Department of Medical Laboratory Science and Biotechnology, College of Medical and Health Science, Asia University, Taichung, Taiwan

**Keywords:** *Helminthostatchys zeylanica*, Ugonin P, Osteoclast, Bone metastasis

## Abstract

Bone metastasis is a significant complication in advanced-stage cancers, especially breast and lung malignancies, profoundly influencing prognosis and quality of life. Osteolytic bone metastasis contains multiple interactions between cancer cells and the bone microenvironment, driving osteoclast-mediated bone resorption and deterioration while releasing growth factors that promote tumor progression. Current treatments, including surgery, radiation, and chemotherapy, often result in severe side effects, highlighting the need for effective, targeted therapies. Ugonin P, a natural compound derived from *Helminthostachys zeylanica*, known for its anti-inflammatory and anticancer properties. However, the effects of Ugonin P on osteolytic bone metastasis remain unclear. Our findings demonstrate that Ugonin P inhibits both RANKL-induced and lung and breast cancer-induced osteoclast formation. Bioinformatics analysis revealed that Midkine (MDK), a heparin-binding growth factor known to promote migration, is highly elevated in breast and lung cancer patients and is related with osteoclast formation. We further showed that MDK is involved in cancer-promoted osteoclastogenesis and that Ugonin P suppresses this process by upregulating miR-223-3p expression. Importantly, Ugonin P effectively blocks lung and breast cancer-facilitated osteolytic bone metastasis *in vivo*. These findings highlight Ugonin P as a promising therapeutic strategy for treating osteolytic bone metastasis.

## Introduction

Metastatic or secondary bone cancer occurs when cancer migrates from its primary site to other organs, particularly bone, due to its rich blood supply. According to Cancer Statistics 2025, prostate, lung, and colorectal cancer are the most diagnosed cancers in men, while breast, lung, and colorectal cancer are most common in women, with breast cancer alone accounting for 32% of cases 1. Lung cancer & breast cancer has the highest bone metastasis rate at diagnosis (18.05%), followed by liver (6.63%), nasopharyngeal (6.33%), and renal cancer (5.45%). Frequently metastasizes to bone, leading to poor survival and severe complications, as highlighted in a retrospective study analyzing risk factors 2. Approximately 75% of breast cancer and 40% of non-small cell lung cancer patients develop bone metastasis in advanced stages, with an average survival of about 9 months 3, 4. Malignant tumor metastasis involves cells escaping the primary site and proliferating in bone, leading to osteolytic bone metastasis through osteoclast (resorption) and osteoblast (formation) activity 5. This process is regulated by various cells and factors, including immune cells, tumor cells, cytokines, growth factors, and inflammatory mediators released by cancer cells, which promote osteolytic bone resorption 6, 7. The interaction between osteoclasts and tumor cells fosters a more aggressive cancer phenotype, increasing the likelihood of metastasis and bone degradation 8, often resulting in spinal cord compression, pathologic fractures and bone pain, all of which significantly reduce patient survival 9. Current treatments, including radiotherapy, chemotherapy, bisphosphonates, denosumab for pain relief, anti-resorptive medications, and surgery, often have limitations impacting survival 10. Therefore, research is increasingly focused on exploring medicines and targeted therapies to mitigate bone metastasis. Hence, we propose to investigate strategies for treating osteolytic bone metastasis.

Midkine (MDK) is a heparin-binding protein that acts as a growth factor or cytokine, promoting cell proliferation, survival, migration, and gene expression. Recent studies highlight MDK's importance as a biomarker, with significantly elevated expression in nearly twenty different tumors compared to normal tissues 11, 12. MDK is crucial in multi-drug resistance, promoting metastasis through mechanisms like overexpression of pro-proliferative factors, promotion of epithelial-mesenchymal transition (EMT), NF-κB activation via Notch 2 receptor interaction, and angiogenesis 13, 14. It also contributes to mitogenesis, anti-apoptosis, and the regulation of antitumor immune responses, particularly linked to chemoresistance in gastric, ovarian, lung and breast cancers 11, 15-18. Increased MDK expression in non-small cell lung cancer (NSCLC) is related with malignant traits and influences breast cancer progression through the NF-κB-NR3C1 pathway, also protecting cancer cells from cannabinoid and doxorubicin treatments. While MDK promotes cancer cell motility, its function in cancer-mediated bone metastasis remains a mystery, suggesting it could be a potential target for remedying osteolytic bone metastasis.

*Helminthostachys zeylanica* (L.) Hook. (HZ), a medicinal plant from Ophioglossaceae family, is native to Southeast Asia 19. The roots of *Helminthostachys zeylanica* (L.) Hooks. have long been applied for inflammation and exhibit different pharmacological properties, including anti-inflammatory, antioxidative, and neuroprotective effects 20-22. Ugonin A-L. derived from HZ, exhibits potent bioactivity against various diseases by exerting antioxidant effects, safeguarding osteoblasts from cellular damage, and inhibiting melanogenesis in melanoma cells 23. Research shows *H. zeylanica* promotes apoptosis in gastric cancer cells via Bax, Bcl2 and caspase 3/7 regulation, COX-2 action, and cleaved poly (ADP-ribose) polymerase mechanisms 24. Additionally, an ethyl acetate-soluble extract from its rhizomes effectively inhibits CD24-/lowCD44+ breast cancer stem cells, while Ugonin L promotes cell death in mature osteoclasts and inhibits RANKL-induced MAPKs and NF-κB activation 25, 26. Furthermore, Ugonin P emerges as a promising therapeutic candidate for arthritis by restoring cartilage homeostasis through miRNA regulation and MAPK pathway activation 27. Ugonin V demonstrated significant potential in suppressing chondrosarcoma metastasis by regulating cathepsin V via miR-4799-5p 28. Furthermore, Ugonin P from *H. zeylanica* inhibits DPP-4-mediated cell migration and invasion in lung cancer cells, enhancing miR-130b-5p expression and disrupting the RAF/MEK/ERK pathway 29. However, the role of Ugonin P in osteolytic bone metastasis remains unexplored. In this study, we confirmed that cancer cells promote bone metastasis through increasing osteoclast formation, which can be inhibited by Ugonin P. We also determined that MDK is highly expressed in lung and breast cancer patients and is related with osteoclast differentiation. Furthermore, we discovered that Ugonin P inhibits MDK-mediated osteolytic bone metastasis by upregulating miR-223-3p *in vitro* and *in vivo*.

## Materials and methods

### Materials

Ugonin P was supplied by Dr. Chih-Chuang Liaw from National Sun Yat-sen University in Taiwan and prepared according to the previous report 29. A TRIzol kit was purchased from MDBio Inc. (Taipei, Taiwan). Human recombinant RANKL was acquired from PeproTech, in (Rocky Hill, NJ, USA). The MDK (SC-46701) and GAPDH antibodies (SC-365062) were sourced from Santa Cruz, CA, USA. The MTT solution, ((3-(4,5-dimethylthiazol-2-yl)-2,5-diphenyltetrazolium bromide) buffer was bought from Sigma-Aldrich (St. Louis, MO, USA). Additional reagents were procured from Sigma Aldrich in (St. Louis, MO, USA) 30, 31. Primer and miRNA inhibitor sequences are listed in the supplementary Table S1, S2.

### Cell culture

The murine RAW264.7 macrophage cell line was obtained from the bioresource collection and Research Center (Hsinchu, Taiwan) and was used as an osteoclast precursor cell line, given its ability to differentiate into osteoclasts in response to RANKL stimulation. The human lung cancer cell lines, CL1-5 and A549 are commonly used models for lung cancer metastasis and progression. The human MDA-MB-231 and 4T1 breast cancer cell lines are highly aggressive triple-negative breast cancer (TNBC) models, with 4T1 is a syngeneic murine model widely used for studying spontaneous metastasis. RAW264.7 cells were cultivated in a DMEM commercial medium (Gibco, Waltham, MA, USA) containing 10% fetal bovine serum (FBS; Gibco, Waltham, MA, USA). CL1-5, A549, and MDA-MB-231 cells were grown in DMEM medium (Gibco, Waltham, MA, USA) supplemented with 10% FBS. 4T1 cells were cultured in RPMI (Gibco, Waltham, MA, USA), supplemented with 10% FBS. All cells were incubated at 37°C in a humidified atmosphere with 5% CO₂ and were passaged upon reaching 80% confluency 29, 32.

### MTT assay

RAW264.7, CL1-5, MDA-MB-231 cells were treated with varying concentrations of Ugonin P (0.1, 0.3, 1, and 3 µM) for 24 hours and then exposed to MTT solution for 2 hours followed by DMSO to facilitate the detection using a microplate reader at a wavelength of 540 nm (BioTek, Winooski, VT, USA) 29.

### Osteoclast Differentiation

RAW264.7 cells were treated with RANKL (50 ng/mL) and various concentrations of Ugonin P. On day five, following established protocols, multinucleated cells were identified as mature osteoclasts using tartrate-resistant acid phosphatase (TRAP) staining 31, 32.

### Immunofluorescence staining

Cells were fixed with 3.7% formalin for 30 minutes, permeabilized using 0.1% Triton X-100, and blocked with 1% BSA for 30 minutes. Subsequently, they were incubated with -Fluor® 488 (Thermo Fisher Scientific, UK) to visualize F-actin following manufacturer recommendation. Nuclei were stained with DAPI for 15 minutes, and immunofluorescence was analyzed using a Carl Zeiss fluorescence microscope.

### GEO database

Gene expression datasets were retrieved from the GEO database, including GSE126548 (lung cancer), GSE191230 (breast cancer), and GSE178196 (osteoclast differentiation). These datasets were selected based on their relevance and statistical significance (*p*-value). The expression profiles were integrated to identify commonly upregulated genes, resulting in 11 shared genes across lung cancer, breast cancer, and osteoclast differentiation. Gene expression analysis was performed using GraphPad Prism to compare levels between normal, cancer, metastasis, and osteoclast groups. MiRNA data were obtained from TargetScan, microT-CDS, and miRDB databases for further analysis of miRNA regulation.

### Quantitative real-time PCR

RNA was isolated from CL1-5 and MDA-MB-231 cells and transcribed to cDNA (100 ng) using the MMLV RT kit (Invitrogen, Carlsbad, CA, USA). For miRNA, cDNA was synthesized from total RNA using oligonucleotide primers and the Mir-X™ miRNA First-Strand Synthesis Kit (Takara, Mountain View, CA, USA). qPCR was performed using the StepOnePlus system following a defined protocol 29.

### Western blotting

Total cell lysates were obtained and quantified using RIPA lysis buffer, followed by the Pierce™ BCA Protein Assay Kit to determine protein concentration. Proteins were resolved by SDS-PAGE and transferred to Immobilon® PVDF membranes. Membranes were blocked with 3% BSA for 1 hour, then incubated overnight with primary antibodies (1:1000) and subsequently with secondary antibodies. Protein bands were visualized using the ImageQuant™ LAS 4000 (GE Healthcare, Little Chalfont, UK) 33, 34.

### Enzyme-linked Immunosorbent assay (ELISA)

Human CL1-5 and MDA-MB-231 cells (2 × 10⁶) were cultured. After 24 hours, the cells were treated with Ugonin P (3 µM) or transfected with MDK plasmid for overexpression (noted as OE MDK). Following 24 hours of treatment and transfection, the cancer cell-conditioned medium (CC-CM) was collected for further analysis 35. MDK level in the CC-CM was quantified using an ELISA kit (Calalog no: DY258, R&D Systems, MN, USA) following the manufacturer's protocol. Briefly, samples were incubated in 96-well plate pre-coated with the capture antibody, followed by the detection antibody and substrate solution. The optical density was measured at 450 nM using a microplate reader.

### Osteolytic bone metastasis *in vivo* model

A549 and MDA-MB-231 Luc cells (1 × 10⁵) were injected into the right tibia of 4-week-old nude mice obtained from BioLASCO Taiwan Co., Ltd. (Taipei, Taiwan). After one week to allow tumor stabilization, the mice were intraperitoneally administered Ugonin P (15 mg/kg) for four weeks. Tumor progression was monitored weekly using IVIS® Spectrum imaging. At the end of the treatment period, all mice were humanely sacrificed, and tumor-induced bone erosion was evaluated by X-ray radiography. Tumor tissues were collected and stained with hematoxylin and eosin (H&E; Rapid Science Co., Ltd., Taiwan) for histopathological analysis. Additionally, TRAP staining was performed to assess osteoclast activity in tibial specimens 32. The Institutional Animal Care and Use Committee of China Medical University approved the animal study.

### Immunohistochemistry

Tumor tissues obtained from the *in vivo* bone metastasis model were stained using the MDK antibody. The staining intensity was evaluated and quantified based on the following scoring criteria: 0 - No staining, 1 - Weak staining, 2 - moderate staining, 3 - strong staining following the methodology detailed in our previous study 35.

### Statistics

Data are presented as mean ± standard deviation (S.D.). Statistical analysis was performed using Student's t-test or one-way ANOVA with Bonferroni correction (GraphPad Prism 8.2). A *p*-value < 0.05 was considered significant.

## Results

### Ugonin P inhibits RANKL-induced osteoclast formation

**Figure 1:**
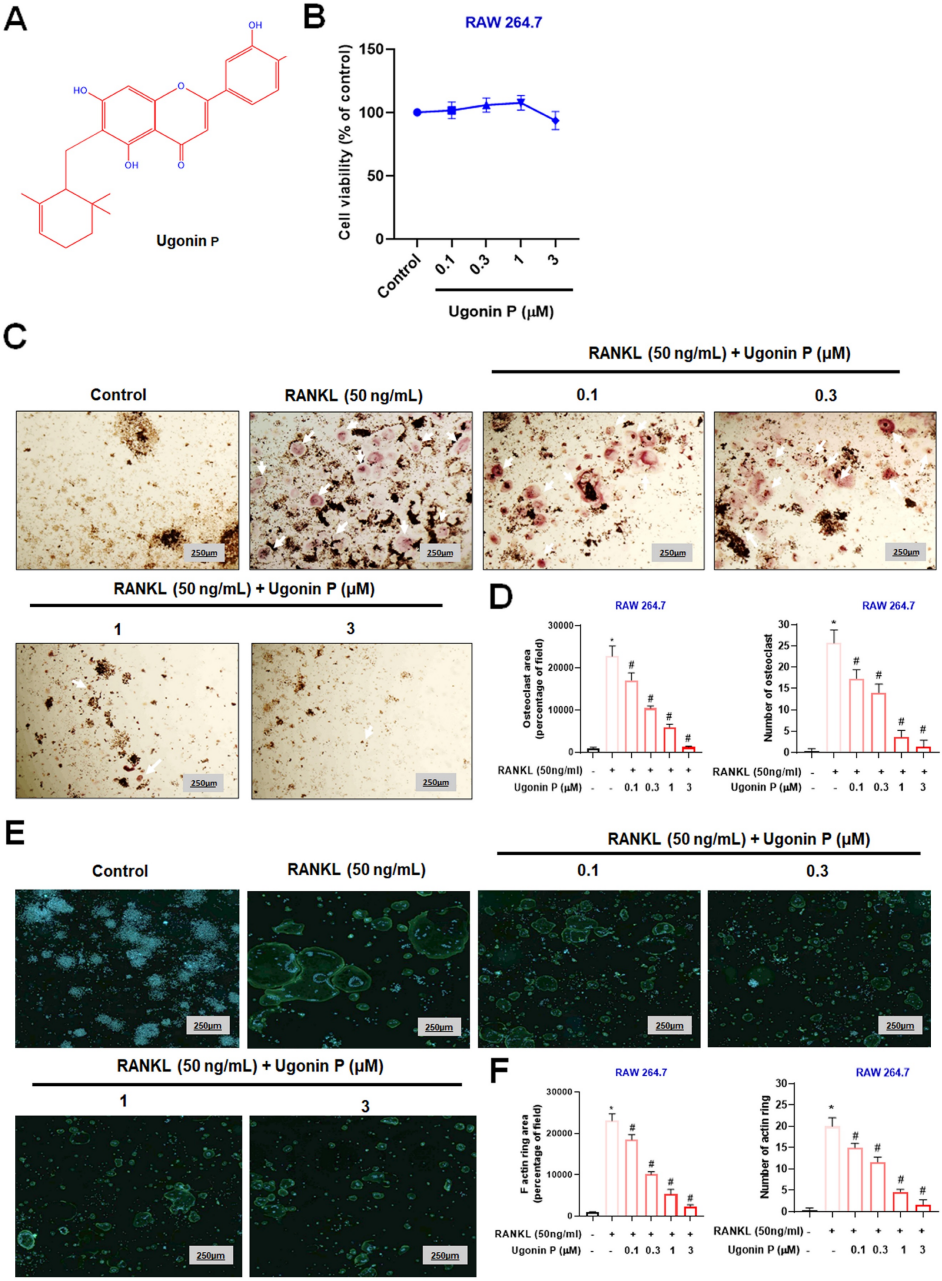
** Ugonin P inhibits RANKL-induced osteoclast formation.** (A) Ugonin P chemical structure. (B) RAW264.7 cells were treated with Ugonin P (0, 0.1, 0.3, 1, 3 µM) for 24 hours, with cell viability assessed using an MTT assay. RAW264.7 cells were treated with RANKL (50 ng/ml) and Ugonin P (0, 0.1, 0.3, 1, 3 µM) for 5 days and stained with TRAP (C-D) (white arrow indicates osteoclasts) and F-actin ring formation (E-F). F-actin (green) and DAPI (blue). ImageJ software quantified the number of positively stained cells or mature osteoclast area. n=3 per group. *p < 0.05 compared with the control group. #*p* < 0.05 compared with the RANKL-treated group.

Osteoclast-mediated bone resorption is a critical mediator in osteolytic bone metastasis, and it is well-established that RANKL promotes osteoclast differentiation in RAW264.7 macrophage cells 31, 34. To evaluate the effects of Ugonin P, we examined its impact on the viability of RAW264.7 cells (Fig. 1A-B) 29. Results confirmed that Ugonin P did not affect cell viability. We then assessed its effect on RANKL-induced osteoclast differentiation by treating RAW264.7 cells with RANKL (50 ng/ml) and varying concentrations of Ugonin P for five days. TRAP staining showed that Ugonin P reduced osteoclast number and area compared to the RANKL-treated group (Fig. 1C-D). F-actin ring formation assays indicated that Ugonin P significantly decreased the functional activity of osteoclasts, as evidenced by reduced F-actin ring formation (Fig. 1E-F). These results show that Ugonin P blocks osteoclast formation without affecting cell viability.

### Ugonin P inhibits cancer cell-mediated osteoclast formation

**Figure 2:**
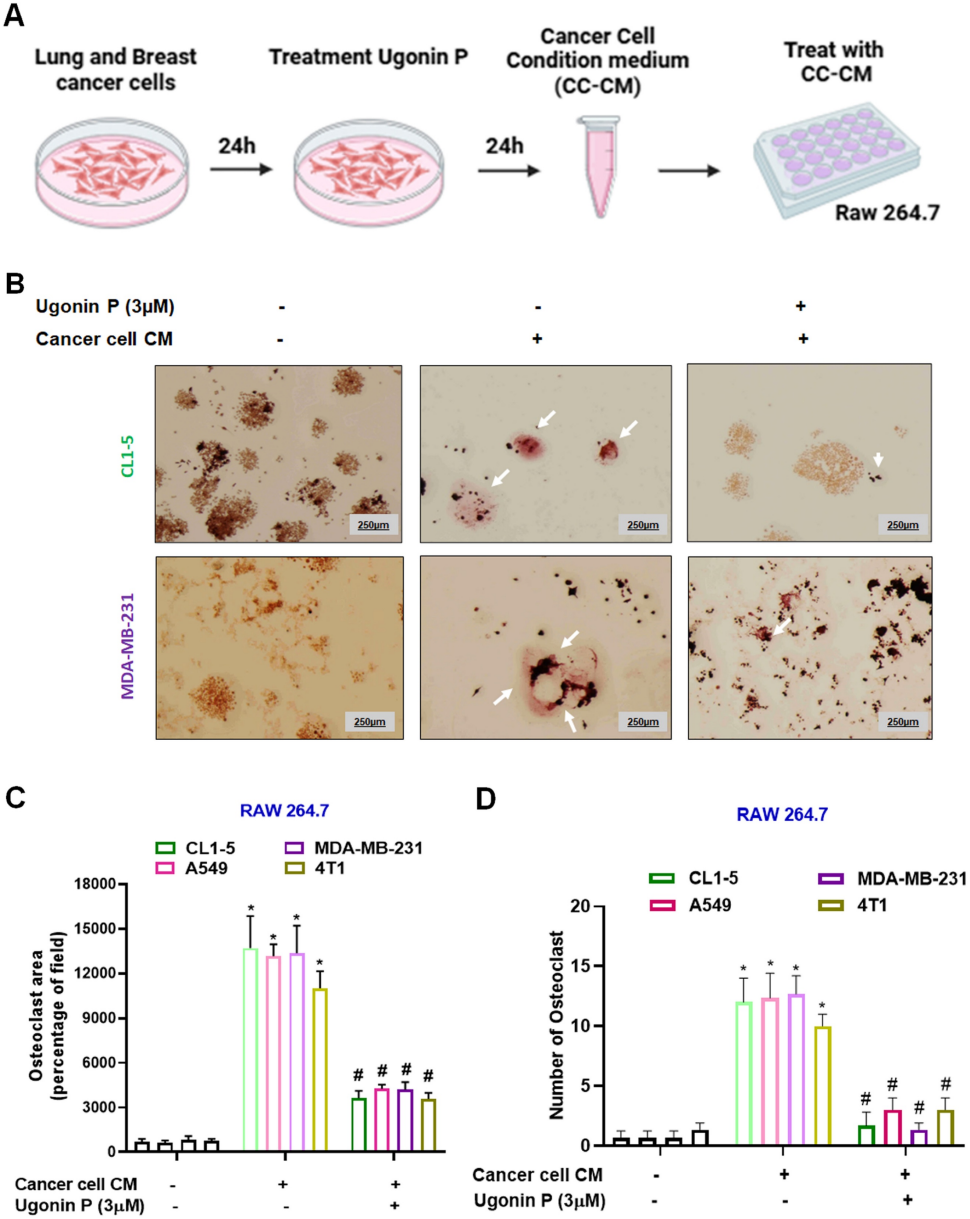
** Ugonin P inhibits cancer-promoted osteoclast formation.** (A) Steps followed to collect cancer cell-conditioned medium (CC-CM). The CC-CM was subsequently collected and applied to RAW264.7 cells, which were then incubated for 5 days. (B-D) Staining of TRAP in RAW264.7 cells treated with CC-CM (white arrow indicates osteoclasts). ImageJ software quantified the number of positively stained cells or mature osteoclast area. n=3 per group. *p < 0.05 compared with the control group. *#p* < 0.05 compared with the RANKL-treated group.

Tumor-secreted factors promote osteoclast differentiation, contributing to osteolytic lesions 36. We then evaluated the effects of osteolytic lung cancer (A549 and CL1-5 cells) and breast cancer (MDA-MB-231 and 4T1 cells) on osteoclastogenesis. Lung and breast cancer cells (CL1-5 and MDA-MB-231) were seeded in 6 well plate. After 24 hrs, the cells were treated with Ugonin P (3 µM) for 24 hrs, and the conditioned medium from the respective groups was collected for further analysis (Fig. 2A). Conditioned medium from cancer cells (CC-CM) was presented to enhance osteoclast formation in RAW264.7 cells. However, treatment of cancer cells with Ugonin P significantly blocked CC-CM-induced osteoclast formation (Fig. 2B-D, Supplementary Fig. S1). These results indicate that Ugonin P effectively suppresses cancer-induced osteoclast formation.

### Ugonin P mitigates cancer-mediated osteoclast formation through inhibiting MDK production

**Figure 3:**
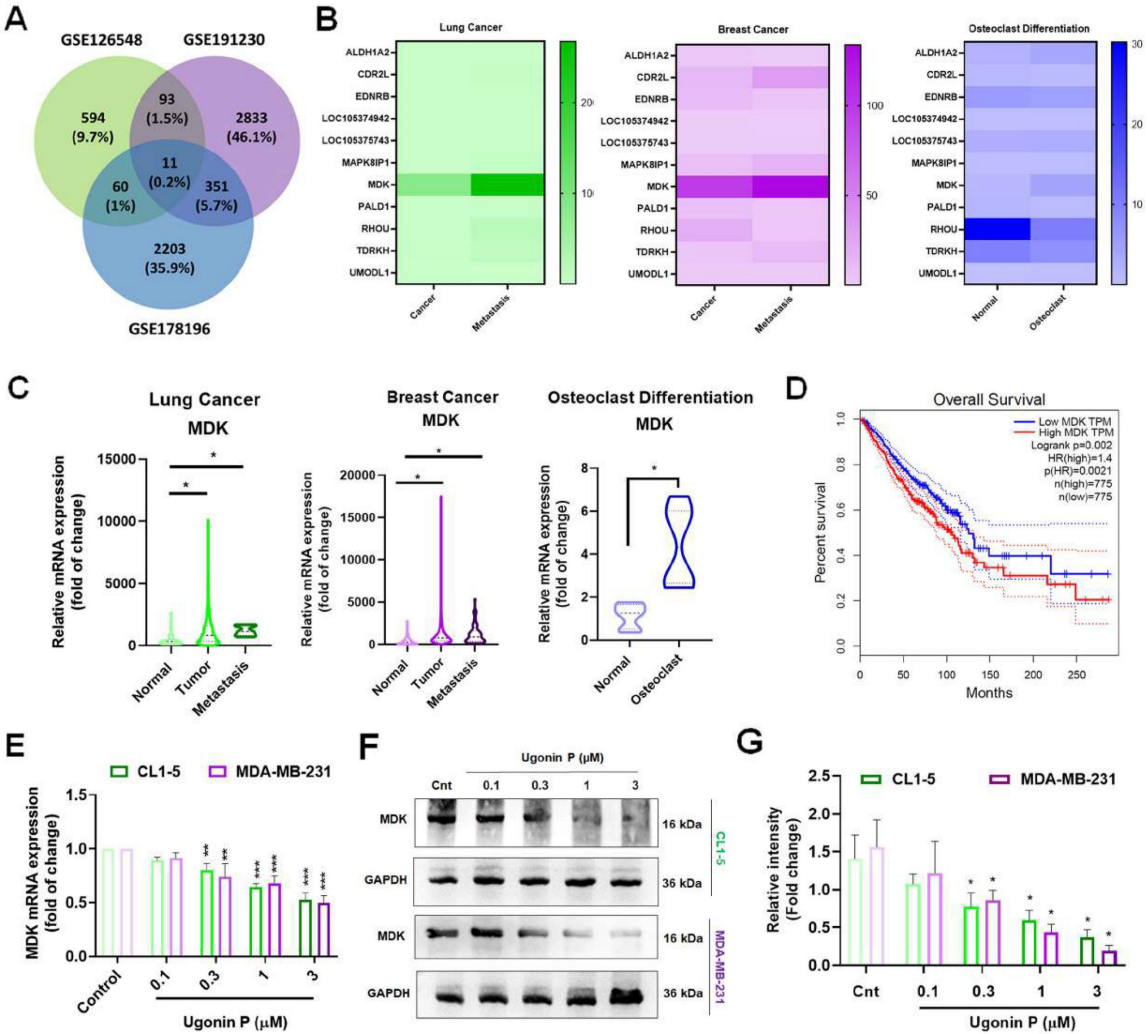
** Ugonin P mitigates MDK expression in cancer cells.** (A) Venn diagram showing the intersection analysis of the GEO database datasets (GSE126548, GSE191230, and GSE178196), revealing 11 potential genes. (B-C) Heatmap and KM Plot analysis of the potential genes across the datasets demonstrates MDK expression in lung cancer, breast cancer, and osteoclast differentiation. (D) Survival analysis of MDK expression in lung and breast cancer patients using the GEPIA database. (E-G) CL1-5 and MDA-MB-231 cells were treated with Ugonin P (0.1,0.3,1,3 µM) for 24 hours. MDK mRNA and protein levels were analyzed via qPCR and western blotting. n=3 per group. *p < 0.05 compared with the control group; **p < 0.01 compared with the control group. ***p < 0.001 compared with the control group.

Cancer cells secrete factors that promote osteolytic bone metastasis 37, 38. Therefore, to identify the key factors in promoting cancer bone metastasis, we analyzed the GEO database for highly metastatic lung and breast cancers alongside osteoclast-related genes which revealed 11 genes are associated with cancer-mediated metastasis, with MDK showing significantly elevated expression, correlating with poor patient survival (Fig. 3A-D). MDK, a heparin-binding growth factor secreted by tumors and is found in soluble form in the tumor microenvironment 11, 39, was further investigated for its inhibitory effect by Ugonin P. To determine the cytotoxic effects of Ugonin P, we utilized concentrations previously established in our study 30.

**Figure 4:**
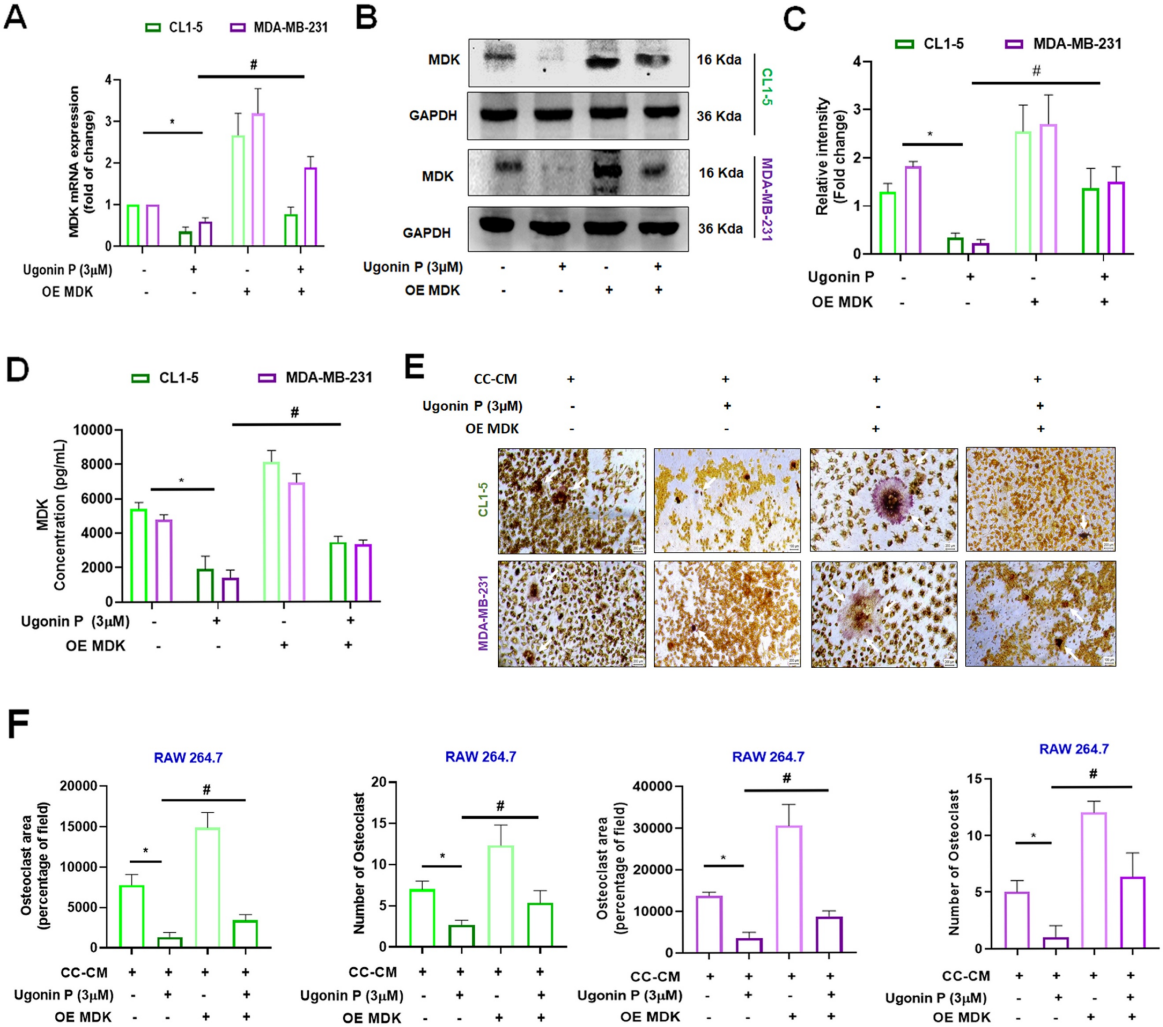
** MDK is involved in Ugonin P-inhibited osteoclast formation.** (A-D) CL1-5 and MDA-MB-231 cells were transfected with the MDK plasmid (Overexpress MDK- OE MDK) and treated with Ugonin P (3 µM) for 24 hours. MDK mRNA and protein levels were analyzed by qPCR, western blotting, and ELISA. (E-F). The CC-CM was collected and applied to RAW264.7 cells, which were then incubated for 5 days. Osteoclast area and number were assessed using TRAP assay. n=3 per group. *p < 0.05 compared with the CC-CM group. *#p* < 0.05 compared with the Ugonin P-treated group.

Cell viability assays revealed that 3 µM was non-toxic to the cells, while higher concentrations exhibited cytotoxicity. The IC50 value was determined to be 25.38 µM in CL1-5 and 44.56 µM in MDA-MB-231 cell lines (Supplementary Fig. S2A-B). Our results demonstrated that Ugonin P effectively suppressed MDK mRNA and protein expression in CL1-5 and MDA-MB-231 in a dose-dependent manner (Fig. 3E-G). To investigate the role of MDK in lung and breast cancer cell-mediated osteoclast formation, CL1-5 and MDA-MB-231 cells were transfected with an MDK plasmid, and mRNA and protein levels were subsequently analyzed. As expected, overexpression of MDK in cancer cells led to increased mRNA and protein production of MDK (Fig. 4A-C). Notably, Ugonin P inhibited MDK expression, and the CC-CM-facilitated osteoclast formation was reversed by MDK overexpression (Fig. 4E-F). These findings indicate that Ugonin P suppresses cancer-mediated osteoclast formation by inhibiting MDK production.

### Ugonin P inhibits MDK expression by upregulating miR-223-3p

**Figure 5:**
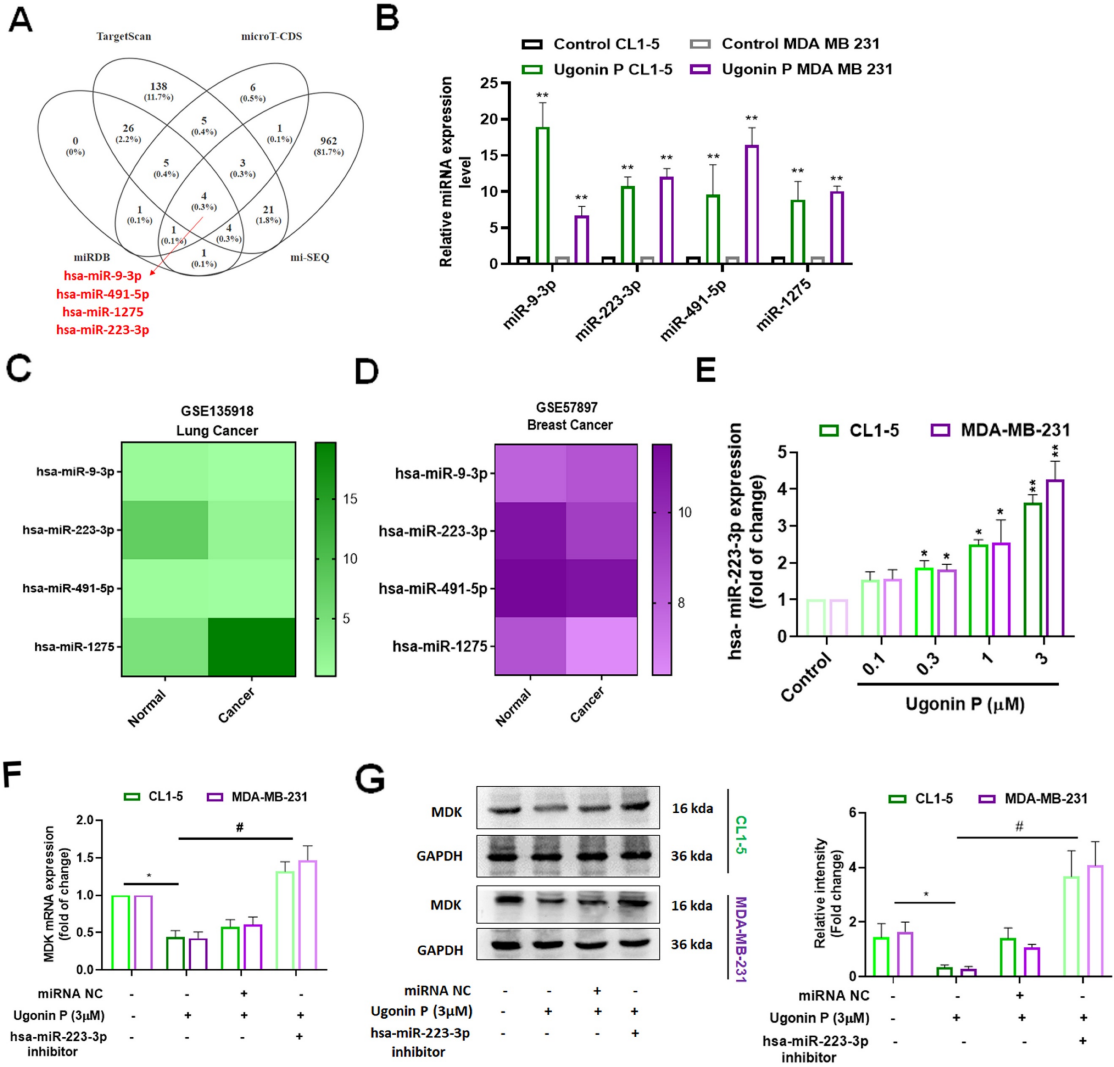
** Ugonin P reduces the expression of MDK by enhancing the levels of miR-223-3p.** (A) A comprehensive analysis of publicly available miRNA databases was conducted to identify miRNAs targeting MDK. (B) qPCR analysis of miRNA expression in CL1-5 and MDA-MB-231 cells treated with Ugonin P (3 µM). (C-D) Heatmap showing differentially expressed miRNAs from the GSE 135918 and GSE 57897 dataset. (E) Cells were treated with Ugonin P for 24 hours, the MDK expression was examined by qPCR. (F-G) Cells were transfected with a miR-223-3p inhibitor and subsequently treated with Ugonin P (3 µM) for 24 hours, the MDK expression was examined by qPCR and western blotting. n=3 per group. *p < 0.05 compared with the control group. **p < 0.01 compared with the control group. *#p* < 0.05 compared with the Ugonin P-treated group.

miRNAs control gene expression post-transcriptionally by binding to specific mRNAs, making them pathological biomarkers and pharmacological targets in cancer 40, 41. We utilized publicly available databases (miRDB, TargetScan, and microT-CDS) and our previously reported Ugonin P-treated lung cancer miRNA sequencing data 30 to identify potential miRNAs targeting MDK. Our analysis revealed that miR-223-3p, miR-9-3p, miR-491-5p, and miR-1275 target MDK (Fig. 5A). Ugonin P significantly upregulated the expression of these miRNAs (Fig. 5B). However, GEO database analysis indicated that miR-223-3p, but not miR-9-3p, miR-491-5p, or miR-1275, is downregulated in both lung and breast cancers compared to normal tissues (Fig. 5C-D). Additionally, Ugonin P enhanced miR-223-3p expression in a dose-dependent manner (Fig. 5E). Transfection with a miR-223-3p inhibitor blocked the suppressive effects of Ugonin P on MDK mRNA and protein levels (Fig. 5F-G). Therefore, Ugonin P inhibits MDK production in cancer cells by upregulating miR-223-3p synthesis.

### Ugonin P inhibits osteolytic bone metastasis *in vivo*

**Figure 6:**
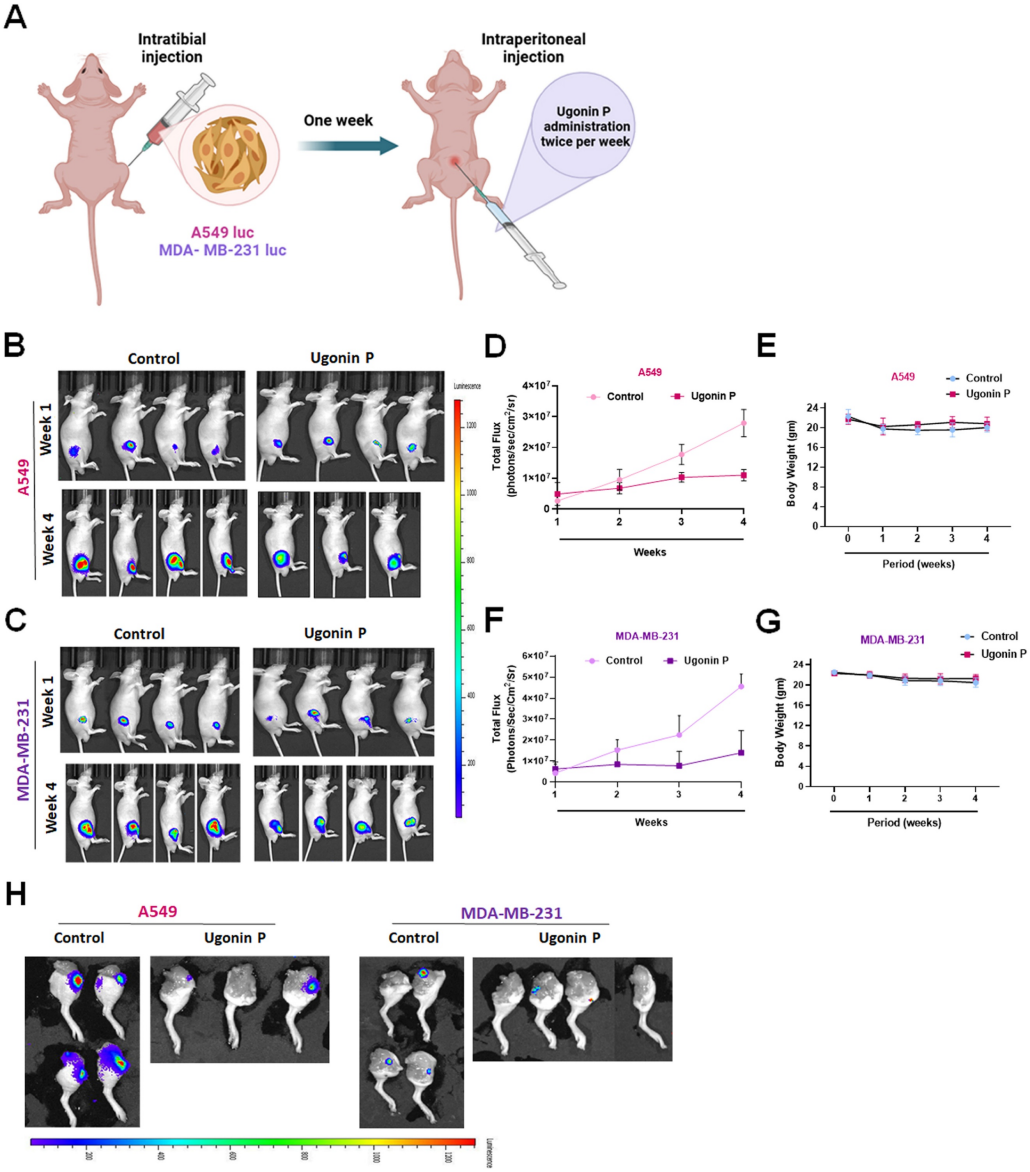
** Ugonin P inhibits osteolytic bone metastasis *in vivo*.** (A) Intra tibial injection of A549 and MDA-MB-231 cells into nude mice followed by intraperitoneally treated with Ugonin P (15 mg/kg) for alternative days for 4 weeks. (B-C) Representative IVIS images of bone metastasis at 1 and 4 weeks. (D-G) Quantification of emitted photons from each tumor by using IVIS and Mouse body weight was monitored three times a week over the four weeks. (H) After four weeks of Ugonin P treatment, all mice were humanely euthanized. The dissected legs from both the control and Ugonin P-treated groups were evaluated for tumor luminescent intensity to assess the treatment's effects. n= 4 mice per group.

**Figure 7:**
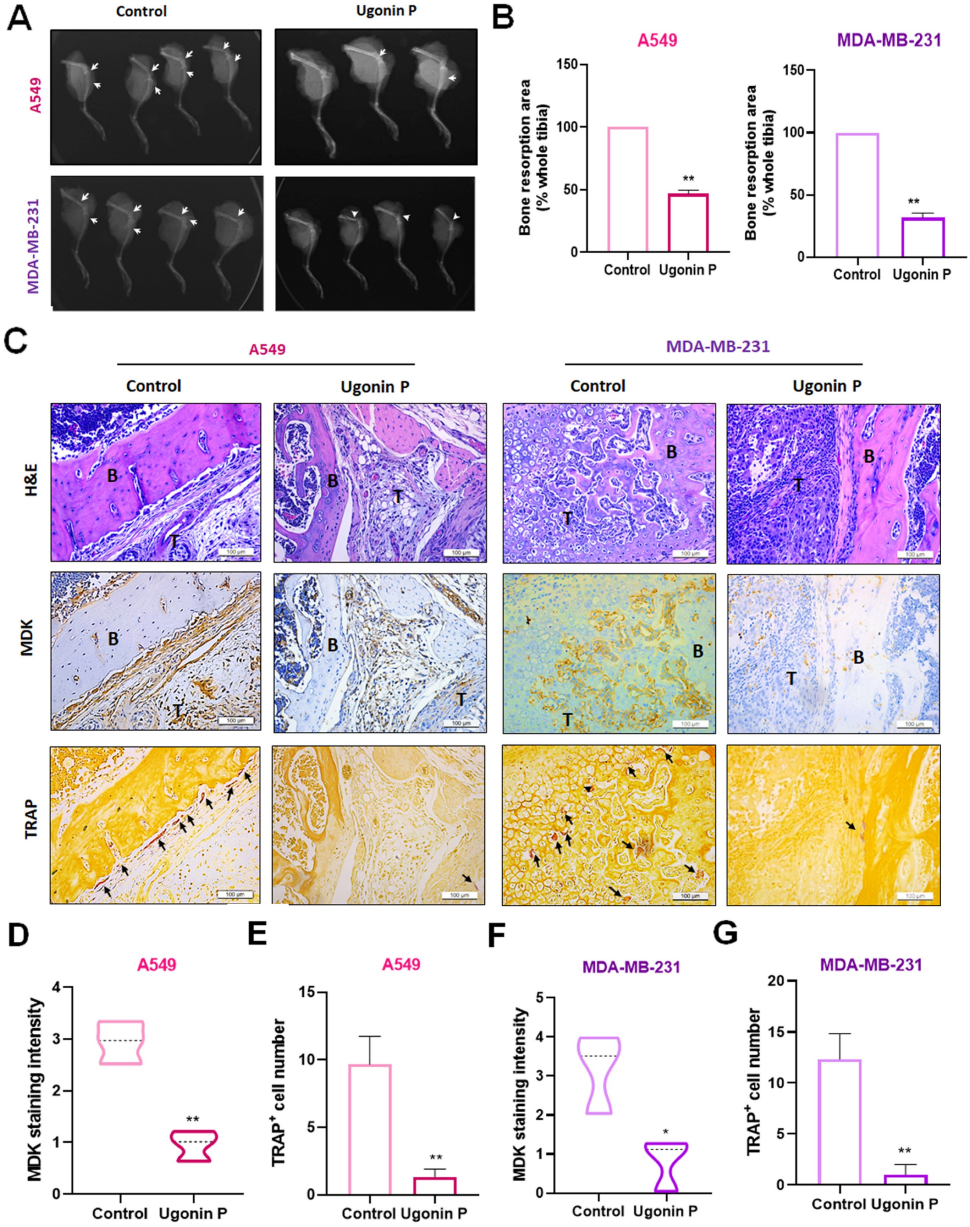
** Ugonin P reduces cancer-promoted MDK expression and osteoclast number *in vivo*.** (A-B) Representative X-ray images of bone erosion in dissected legs. (C) Representative H&E, immunohistochemical images of MDK and TRAP-positive staining images of mouse leg bones. (D-G) The quantitative data for (C). n=3 per group. *p < 0.05 compared with the control group. **p < 0.01 compared with the control group.

To investigate the role of Ugonin P in inhibiting osteolytic bone metastasis *in vivo*, A549 and MDA-MB-231 cells were injected intratibially into nude mice, followed by intraperitoneal administration of Ugonin P (Fig. 6A). Tumor growth in the tibia was significantly slowed by Ugonin P treatment after 4 weeks, as confirmed by *in vivo* bioluminescence imaging, without affecting body weight (Fig. 6B-H)**.** X-ray imaging and H&E analysis showed that Ugonin P inhibited tumor growth from the tibia to the femur and reduced bone erosion in both the A549 and MDA-MB-231 groups (Fig. 7A-C). Immunohistochemistry and TRAP staining revealed lower MDK expression and a reduced number of osteoclasts in the Ugonin P-treated group (Fig. 7C-G). These findings suggest that Ugonin P suppresses lung and breast cancer-promoted osteolytic bone metastasis *in vivo*.

## Discussion

Bone metastases are among the most common types of metastatic tumors, significantly reducing patient's lifespan and quality of life. These metastases result from osteolytic factors secreted within the tumor microenvironment, which disrupt the balance between osteoclast and osteoblast activity, driving osteolytic bone metastasis 42. Among these factors, RANKL plays a central role in bone loss by increasing bone resorption and the collapse of bone tissue 43, 44. *H. zeylanica* has been documented to reduce pro-inflammatory cytokines in macrophage cells 45. Our previous study confirmed that Ugonin P mitigates lung cancer invasion and migration 30.

However, the role of Ugonin P in osteolytic bone metastasis remains largely unclear. In this study, our results revealed that Ugonin P inhibits RANKL-induced osteoclastogenesis. Moreover, osteoclast formation promoted by conditioned medium from both lung and breast cancer cells was significantly suppressed by Ugonin P treatment. Importantly, *in vivo* experiments showed that lung and breast cancer-facilitated osteoclast formation and bone erosion were markedly reduced following Ugonin P treatment. These findings highlight the potential of Ugonin P to mitigate osteoclast-driven bone destruction, suggesting its therapeutic value in managing osteolytic bone metastases.

MDK plays a multifunctional role beyond metastasis and, along with pleiotrophin, constitutes a structurally unique family of heparin-binding growth factors 46. MDK is crucial for embryonic development, tissue repair, and neuronal growth. While MK expression is low in the kidney, it is highly expressed in various solid tumors, including oral, esophageal, colorectal, prostate, lung, breast, cervical, brain cancers, and neuroblastoma 47. In cancer, overexpression of MDK induces tumor progression by promoting cell proliferation, survival, metastasis and angiogenesis, facilitating the development of new blood vessels that supply nutrients to tumors 48. MDK functions as a mitogenic, proinflammatory, and proangiogenic factor and is closely linked to cancer migration and metastasis through major EMT pathways 11, 49. In lung cancer, previous studies have shown a strong association between elevated MDK expression (at both mRNA and protein levels) and malignant status, as well as poor prognosis in NSCLC patients 50. Additionally, MDK has been identified as a minimally invasive biomarker for NSCLC detection and development 51. In breast cancer, MDK knockdown inhibited cell growth and invasion by regulating the NF-κB-NR3C1 pathway 52. Moreover, MDK enhances the pro-metastatic effects of IFN-γ by inducing the EMT program 53. Beyond its role in cancer, MDK is also implicated in inflammatory responses, with elevated levels observed following fractures in estrogen-deficient mice and postmenopausal women with fractures 54. In our current study, we conducted a cross-analysis of the GEO database for lung cancer, breast cancer, and osteoclast formation, identifying MDK as the most likely regulator of lung and breast cancer bone metastasis. We also found that Ugonin P significantly suppresses MDK synthesis in both lung and breast cancer cells. However, overexpression of MDK reversed the Ugonin P-mediated inhibition of CC-CM-induced osteoclast number and area. Therefore, CC-CM containing MDK and MDK-induced osteolytic factors likely contribute to osteoclast formation. Previous studies have confirmed that MDK promotes osteoclastogenesis through both RANKL-dependent and RANKL-independent pathways 55. Eventually, MDK plays a critical role in osteoclastogenesis via both RANKL-dependent and -independent mechanisms. Our findings provide new insights into the role of MDK in regulating osteoclastogenesis. Specifically, Ugonin P blocks cancer-induced osteoclast formation by reducing MDK production.

miRNAs are small non-coding RNA molecules that control gene synthesis by targeting mRNAs through base pairing in their 3′UTR. This interaction leads to mRNA degradation or translational repression, playing a crucial role in both transcriptional and post-transcriptional regulation 40, 41. miRNAs regulate a wide range of cellular functions, such as cell growth, apoptosis, proliferation, survival, angiogenesis, metastatic biomarker identification, and drug resistance 56, 57. By integrating datasets from miRDB, TargetScan, microT-CDS, and our previous sequencing results 30, we identified four potential miRNAs that directly bind to MDK. Among these, miR-223-3p, but not miR-9-3p, miR-491-5p, or miR-1275, is expressed at lower levels in lung and breast cancers compared to normal tissues. Previous studies have reported similar findings, indicating that miR-223-3p is markedly downregulated in LSCC tissues compared to adjacent non-tumoral tissues, with even greater suppression observed in successfully engrafted tumors 58. Furthermore, Ugonin P was found to enhance miR-223-3p expression in lung and breast cancer cells. The use of a miR-223-3p inhibitor reversed the Ugonin P-mediated reduction in MDK production, indicating that Ugonin P decreases MDK expression and osteoclast formation by upregulating miR-223-3p synthesis.

Current cancer treatments, including surgery, chemotherapy, radiotherapy, and immunotherapy, often lead to significant side effects due to their impact on rapidly dividing healthy tissues such as bone marrow and the gut lining 59. Traditional Chinese Medicine (TCM) has emerged as a complementary therapeutic strategy, with compounds like Resveratrol and Curcumin showing promise in inhibiting cancer stem cells and tumor growth 60. Additionally, TCM further reduces resource wastage and strengthens Taiwan's biotech and pharmaceutical research capabilities 61. Ugonin-derived compounds from TCM have been particularly noted for their dual anabolic and anticatabolic effects, promoting bone formation while preventing excessive bone resorption 62. Ugonin compounds, such as Ugonin L, have documented the ability to stimulate osteoblast differentiation and enhance the expression of osteoblast-specific genes in osteoblastic cells 63, 64. Here, we demonstrated the novel functions of Ugonin P in inhibiting cancer-promoted osteoclast formation both *in vitro* and *in vivo*, suggesting its potential for treating osteolytic bone metastasis. However, a key limitation of our study is the lack of micro-CT analysis, which would have provided a more detailed and quantitative assessment of the bone architecture model to help understand the tumor and bone interaction. For clinical application, the chemical structure of Ugonin P requires optimization to enhance potency, aiming to reduce the effective concentration from μM to nM. Further research is also needed to assess its pharmacokinetics, bioavailability, and therapeutic potential in clinical settings where these would enhance the translational potential of Ugonin P as a therapeutic option for osteolytic bone metastasis.

**Figure 8:**
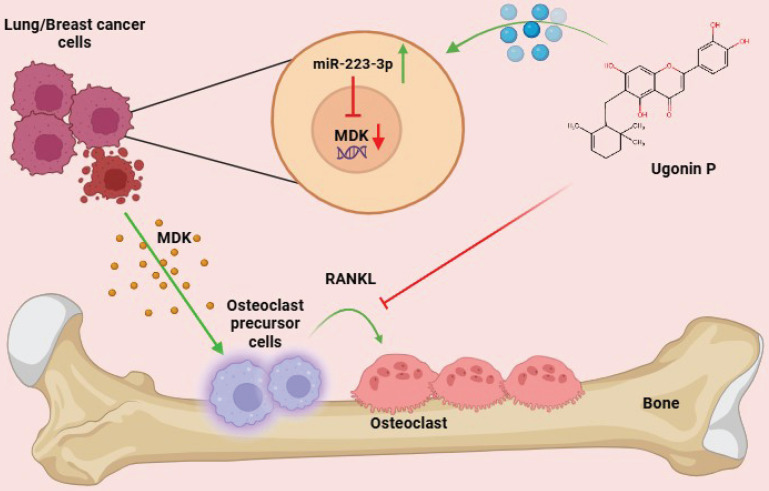
**Schematic representation illustrates the impact of Ugonin P on osteolytic bone metastasis. (The schema was generated utilizing BioRender.com).** Ugonin P inhibits lung and breast cancer-promoted osteoclast formation by reducing MDK production through upregulation of miR-223-3p expression.

In conclusion, Ugonin P effectively inhibits lung and breast cancer-promoted osteoclast formation by reducing MDK production through upregulation of miR-223-3p expression. Importantly, Ugonin P also blocks lung and breast cancer growth in bone and osteolytic areas, inhibiting osteolytic bone metastasis. These findings underscore the potential of Ugonin P as a therapeutic agent for treating osteolytic bone metastases (Fig. 8).

## Supplementary Material

Supplementary figures and tables.
