# Supplementary material for: Ugonin P mitigates osteolytic bone metastasis by suppressing MDK via upregulating miR-223-3p expression
Source: Int J Biol Sci. 2025 May 31;21(8):3740–54. doi: 10.7150/ijbs.111356 (PMC12160927; doi:10.7150/ijbs.111356)
Supplement: Supplementary file 1 — Supplementary figures and tables. [file ijbsv21p3740s1.pdf]

Supplementary Table 1. Primers were used in this study

Gene	Forward	Reverse
MDK	GCTACAATGCTCAGTGCCAGGA	CTTGGCGTCTAGTCCTTTCCC T
GAPDH	AATGGACAACTGGTCGTGGA	CCCTCCAGGGATCTGTTTG
**hsa-miR-9-3p**	TCTTTGGTTATCTAGCTGTATGA	TGGTGTCGTGGAGTCG
**hsa-miR-491-5p**	AGTGGGGAACCCTTCCATGAGG	TGGTGTCGTGGAGTCG
**hsa-miR-223-3p**	TGTCAGTTTGTCAAATACCCCA	TGGTGTCGTGGAGTCG
**hsa-miR-1275**	GTGGGGGAGAGGCTGTC	TGGTGTCGTGGAGTCG
**U6snRNA**	CTCGCTTCGGCAGCACA	AACGCTTCACGAATTTGCGT

Supplementary Table 2. miRNA inhibitor used in this study

miRNA	Sequence
hsa-miR-223-3p inhibitor	UGGGGUAUUUGACAAACUGACA
hsa-miR-223-3p Negative Control	CAGUACUUUUGUGUAGUAGUACAA

Supplementary Figure 1

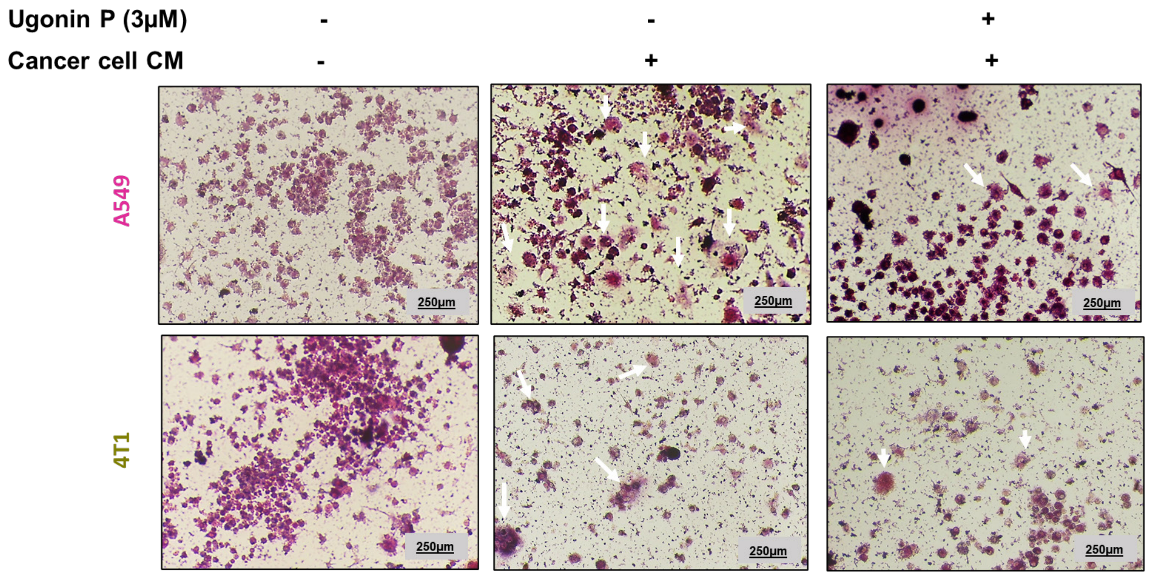

Supplementary Figure 1: Ugonin P inhibits cancer-promoted osteoclast formation. The CC-CM was subsequently collected and applied to RAW264.7 cells, which were then incubated for 5 days. Staining of TRAP in RAW 264.7 cells treated with CC-CM (white arrow indicates osteoclasts). ImageJ software quantified the number of positively stained cells or mature osteoclast area.

Supplementary Figure 2

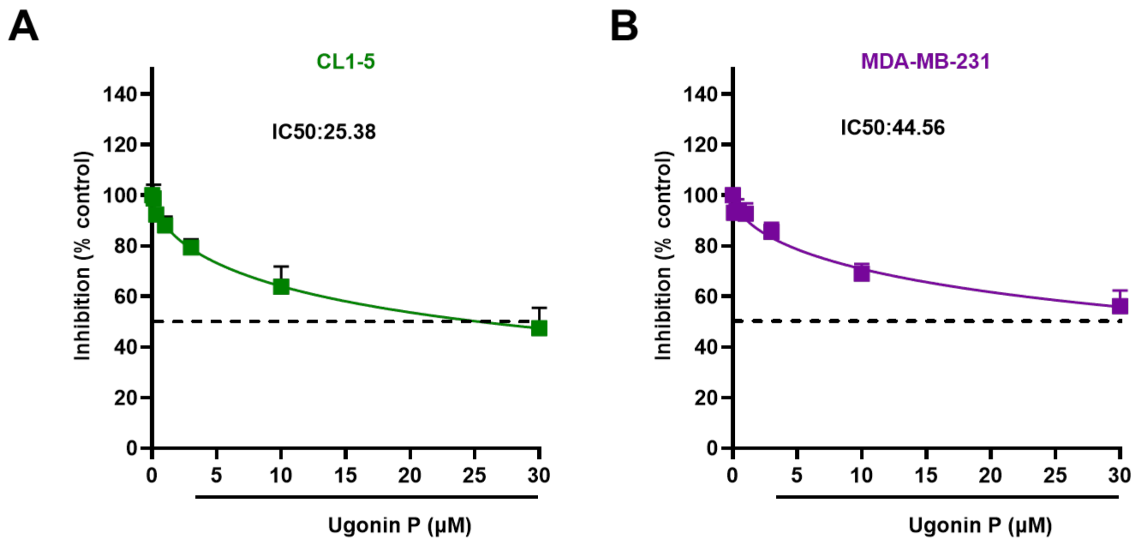

Supplementary Figure 2: IC50 values for Ugonin P in CL1-5 and MDA-MB-231 cells. Cells were treated with increasing concentrations of Ugonin P (0.1-30μM) for 24 hours, and cell viability was measured using the MTT assay. IC50 values were calculated using non-linear regression analysis. Data are shown as mean ± SD from three independent experiments.
